# Drug-tolerant persister cells in cancer: a scoping review of definitions, models, and molecular mechanisms

**DOI:** 10.3389/fonc.2026.1771061

**Published:** 2026-04-29

**Authors:** Jose S. Lopez-Gonzalez, Mario Perez-Medina, Miriam Galicia-Velasco, Dolores Aguilar-Cazares

**Affiliations:** 1 Laboratorio de Investigacion en Cancer Pulmonar, Departamento de Enfermedades Cronico-Degenerativas, Instituto Nacional de Enfermedades Respiratorias “Ismael Cosio Villegas”, Mexico City, Mexico; 2 Department of Research, Asociacion Para Evitar la Ceguera en Mexico IAP, Mexico City, Mexico

**Keywords:** cancer cell plasticity, drug-tolerant persister (DTP) cells, epigenetic reprogramming, metabolic rewiring, non-genetic drug resistance, reversible drug tolerance, therapy-induced adaptation, tumor relapse

## Abstract

Drug-tolerant persistent (DTP) cells have emerged as a reversible, slow-cycling survival state that enables early therapeutic tolerance and underlies the development of stable resistance in all types of cancer. To comprehensively characterize this phenomenon, we conducted a PRISMA-ScR-guided exploratory review across four major databases (PubMed, Scopus, Web of Science, Dimensions), identifying 343 eligible records spanning 2010-2025. In all experimental systems, including 2D cell lines, spheroids, organoids, xenografts, residual disease models, and clinical samples, DTP cells consistently showed survival under high drug concentrations or prolonged exposure, depending on non-genetic adaptive programs, and recovery of proliferative potential and drug sensitivity after treatment cessation. Analysis of the molecular mechanisms revealed a convergence of reversible pathways involving apoptosis escape, quiescence, chromatin remodeling, phenotypic plasticity, metabolic rewiring, downstream survival signaling, and transient programs, such as those of stem cells. These findings support a model in which DTP cells represent an early and plastic node within a broader continuum of resistance, capable of progressing toward genetically fixed resistance through stress-induced mutagenesis. Methodological heterogeneity among studies did not diminish the reproducibility of DTP cells fundamental characteristics but underscored the need for standardized experimental criteria. Notably, the integrated evidence identifies therapeutically exploitable vulnerabilities—epigenetic, metabolic, signaling-based, and plasticity-targeted—that have shown promise in reducing DTP persistence and delaying the development of resistance. This review consolidates current knowledge and provides a mechanistic framework to guide therapeutic strategies that aim to intercept cancer resistance in the earliest and most reversible stages of its development.

## 1 Introduction

The earliest attempts to treat cancer date back to the late nineteenth century, when Heinrich Lissauer administered Fowler’s solution to patients with chronic leukemia, achieving only transient improvement despite its inherent arsenic toxicity (1). During World War II, observations of mustard gas injuries led to the development of nitrogen mustard derivatives, including mechlorethamine, which was the first cytotoxic drug approved by the FDA (2). In the 1960s, Frei, Holland, and Freireich introduced combination chemotherapy based on antibiotic principles, establishing a new therapeutic paradigm for cancer treatment (3). Advances in molecular biology have enabled the development of targeted therapies that have transformed the treatment of several types of cancer. However, despite these successes, therapeutic resistance remains almost universal in patients with advanced cancer. Clinical examples, such as EGFR-mutated lung cancer treated with first-line osimertinib (4), BRAF-mutated melanoma treated with vemurafenib (5), and HER2-positive breast cancer treated with dual HER2 blockade (6), consistently show profound initial responses followed by eventual relapse.

Therapeutic resistance refers to the ability of tumor cells to evade or withstand the effects of drugs. Intrinsic resistance is present from the beginning of therapy. This can arise from pre-existing heterogeneity, plastic cellular states, such as partial epithelial–mesenchymal transition (EMT) or stem-like programs, and basal epigenetic configurations that limit drug sensitivity (7–9). In contrast, acquired resistance develops after initial exposure to the drug, which is typically attributed to secondary mutations or the activation of compensatory pathways. Yet, several studies show early tumor progression without detectable resistance mutations, or progression occurring before such mutations arise (10–12). These observations reveal that early therapeutic escape often involves non-genetic mechanisms, challenging the classical gene-centric model of resistance.

Phenotypic plasticity is central to understanding this phenomenon. Tumor cells can reorganize chromatin, modulate transcription, rewire metabolism, and temporarily reduce biosynthetic activity in response to drug pressure (8, 9, 13). These adaptive transitions decouple viability from proliferation, enabling cells to enter slow-cycling or quasi-dormant states that promote transient drug tolerance (14, 15). The repeated observation of cells surviving lethal drug concentrations, maintaining viability without division, and subsequently regaining sensitivity once therapy is withdrawn cannot be reconciled with traditional clonal selection (16, 17).

In 2010, Sharma et al. identified a reversible chromatin-mediated tolerant state that allowed cancer cells to survive exposure to lethal doses of EGFR inhibitors, introducing the concept of drug-tolerant persister (DTP) cells (15). Since then, numerous studies have explored this notion. DTP cells represent a small subpopulation capable of surviving therapy without acquiring new mutations. They exhibit profound epigenetic reprogramming, transcriptional repression, specific metabolic adaptations, including dependencies on fatty acid oxidation or oxidative phosphorylation, and markedly reduced proliferation (11, 16–18). DTP cells can assume diverse identities, including diapause-like states, partial EMT phenotypes, and neural crest-like transcriptional programs (13, 18). When the drug pressure is removed, these cells regain a proliferative and drug-sensitive phenotype, confirming the reversible nature of this state. However, during therapy, DTP cells act as reservoirs that sustain minimal residual disease and facilitate the subsequent emergence of stable genetic resistance through stress-induced mutagenesis, including APOBEC3A activation, REV1-mediated bypass synthesis, or other error-prone repair processes (18, 19).

The conceptualization of DTP cells was strengthened by their close analogy to bacterial persistence. Since the pioneering work of Bigger, Lewis, and Balaban, it has been recognized that bacterial populations generate a small fraction of non-growing cells that survive lethal antibiotic exposure without harboring resistance mutations (20–23). These bacterial persisters suppress metabolic activity, halt proliferation, and remain viable until the environment becomes permissive. A similar phenomenon occurs in cancer: a minority of tumor cells can enter reversible phenotypic states that permit temporary survival under therapeutic pressure (8, 18). This phenomenon reflects a strategy of phenotypic diversification, in which a small fraction of the population adopts an alternative low-risk state that increases the overall probability of survival in fluctuating environments (21). The parallel between bacterial and cancer persistence reframed early therapeutic tolerance in cancer not as a mutational event but as a reversible adaptive state (18). Together, these findings reshape our understanding of drug resistance. Many tumors do not initially progress due to genetic alterations; instead, they traverse a reversible tolerance phase, represented by DTP cells. This transient state marks the early stage of a broader resistance continuum, preceding the emergence of stable genetic resistance (11). Despite the growing interest in this phenomenon, the field remains conceptually heterogeneous. No consensus exists regarding the conceptual definition of DTP cells, detection methods vary widely, and experimental models differ substantially across studies (18). Therefore, the objective of this study was to map this landscape to identify definitions, experimental models used to study DTP cells, shared characteristics of DTP cells across cancer types, and the molecular mechanisms proposed for their establishment. This, in turn, we believe will contribute to the development of therapeutic strategies targeting drug-tolerant cellular states.

## 2 Methodology

This exploratory review was conducted in accordance with the PRISMA-ScR (Preferred Reporting Items for Systematic Reviews and Meta-Analyses extension for Scoping Reviews) framework (24) and followed a predefined protocol to ensure methodological rigor, transparency, and reproducibility. This review systematically maps the experimental and conceptual literature on drug-tolerant persistent (DTP) cells in cancer and characterizes the range of experimental models used to generate and study these states. It also aims to identify the convergent biological mechanisms underlying reversible therapeutic tolerance and to synthesize emerging therapeutic strategies targeting DTP-associated vulnerabilities.

### 2.1 Search strategy

A comprehensive search was conducted in four electronic databases: PubMed/MEDLINE, Web of Science Core Collection, Scopus, and Dimensions databases. Dimensions was included because several studies describing molecular and transcriptomic mechanisms foundational to the development of the DTP field first appeared on this platform before being indexed in traditional bibliographic databases. This approach is consistent with PRISMA-ScR guidance and updated methodological recommendations for scoping reviews, which support expanding data sources when mapping rapidly evolving scientific domains (24, 25). Specific search strings were developed for each database using combinations of controlled vocabulary (e.g., MeSH, Emtree) and free-text terms covering DTP cells, cancer types, experimental systems, and mechanisms of reversible drug tolerance, following established guidelines for comprehensive literature searches as outlined in the Cochrane Handbook for Systematic Reviews of Interventions (26). The search covered all publications available until September 2025 and imposed no restrictions on the study design.

### 2.2 Eligibility criteria

For this review, drug-tolerant persistent (DTP) cells are defined as a subset of cancer cells that survive exposure to therapeutic agents without developing stable genetic resistance. Eligible studies are those that describe cell populations demonstrating: (i) survival in the presence of drug concentrations that would normally be lethal, (ii) a reversible state of growth arrest or slowed cell cycle, and (iii) recovery of proliferative ability after drug withdrawal. Studies that report irreversible resistance caused by stable genetic changes without evidence of a reversible tolerance state were excluded from this classification.

The selected publications included *in vitro* studies using two-dimensional cell lines or three-dimensional systems, such as spheroids and organoids; *in vivo* models, including xenografts and patient-derived xenografts; and clinical or ex vivo studies evaluating residual disease or drug response in human tumor samples. Studies examining the molecular, transcriptional, epigenetic, metabolic, or phenotypic characteristics of DTP cells were included, as were conceptual reviews that clarified definitions or mechanistic frameworks relevant to DTP biology.

Studies focused exclusively on microbial persistence or antibiotic tolerance, or those unrelated to cancer, were excluded (25). Editorials, conference abstracts, and short communications were also excluded if they lacked methodological details or did not provide extractable data. Preprints were retained only when peer-reviewed versions were unavailable. Studies for which full texts could not be accessed following institutional and open-access retrieval attempts were excluded from further analysis.

### 2.3 Study selection

Three reviewers independently screened all the titles and abstracts (n = 770). Articles considered potentially relevant were thoroughly assessed. Disagreements were resolved through discussion and consensus-building. A total of 343 studies met the eligibility criteria and were included in this review. Of these, 175 were identified as experimental or clinical-experimental studies, and 168 were classified as reviews or conceptual papers. A total of 427 records were excluded at the screening stage due to irrelevance, lack of focus on cancer, or insufficient association with DTP-related phenomena. All the included studies were integrated into the master database and subsequently organized into analytical categories aligned with the results section.

### 2.4 Data extraction and synthesis

A structured analytical matrix was developed to chart the key characteristics of each study, consistent with the established scoping review methodology (27), including cancer type, experimental model, therapeutic agents, exposure conditions, strategies used to induce or identify DTP states, phenotypic and molecular features, evidence of reversibility, and functional validation strategies. Three reviewers independently extracted the data to ensure consistency and accuracy of the results. The extracted variables were harmonized across studies to support the thematic synthesis. Given the heterogeneity of study designs, model systems, and mechanistic endpoints, the synthesis was descriptive and narrative, integrating experimental evidence, molecular mechanisms, and therapeutic implications without altering the citation order or numbering.

### 2.5 Data processing and visualization

**Figure 1:**
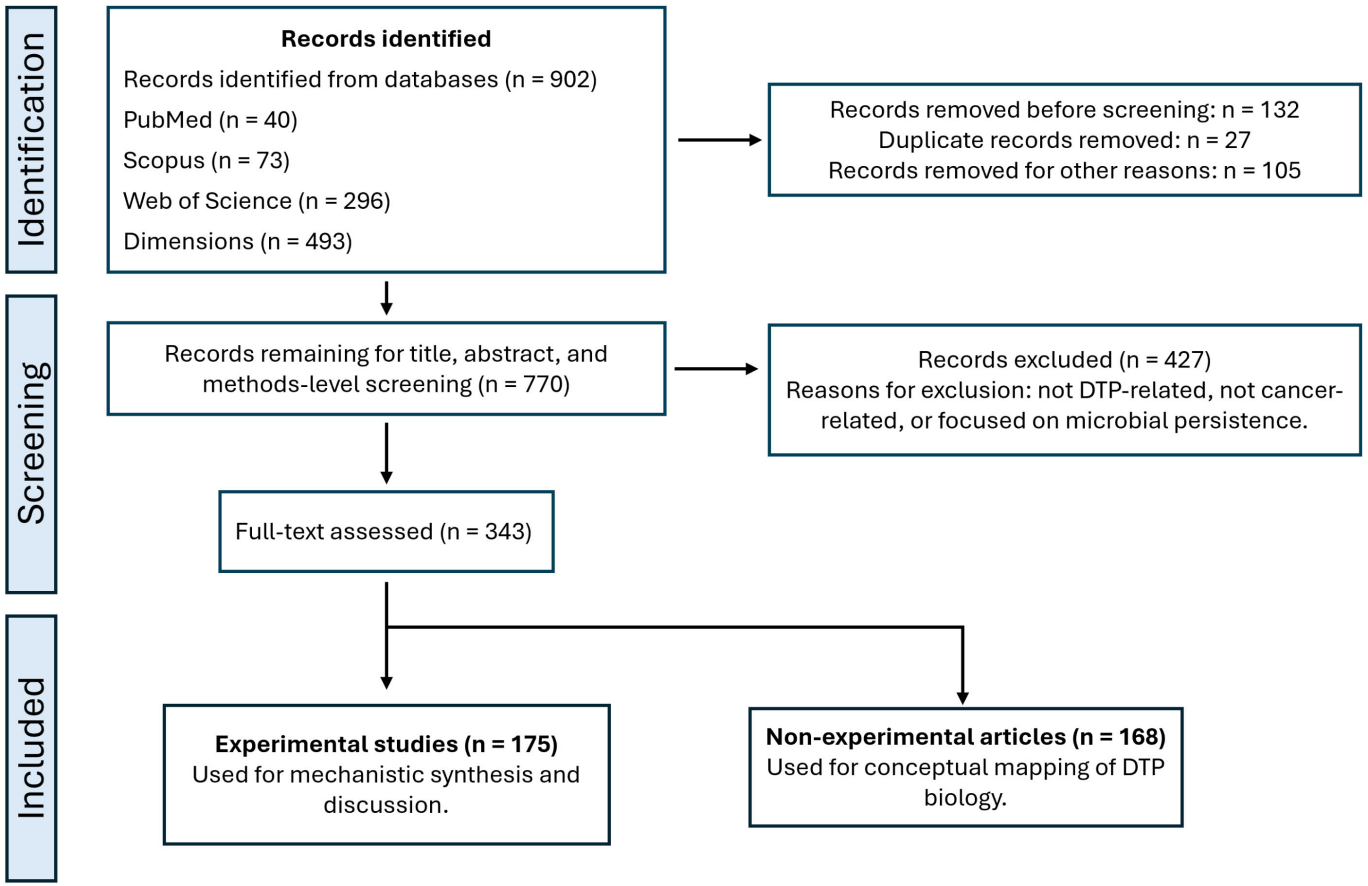
PRISMA-ScR flow diagram of study selection. PRISMA-ScR flow diagram summarizing the identification, screening, eligibility, and inclusion of studies in this scoping review. Records were identified through systematic searches across multiple bibliographic databases, followed by removal of duplicates, title and abstract screening, full-text assessment, and final inclusion according to predefined eligibility criteria.

**Figure 2:**
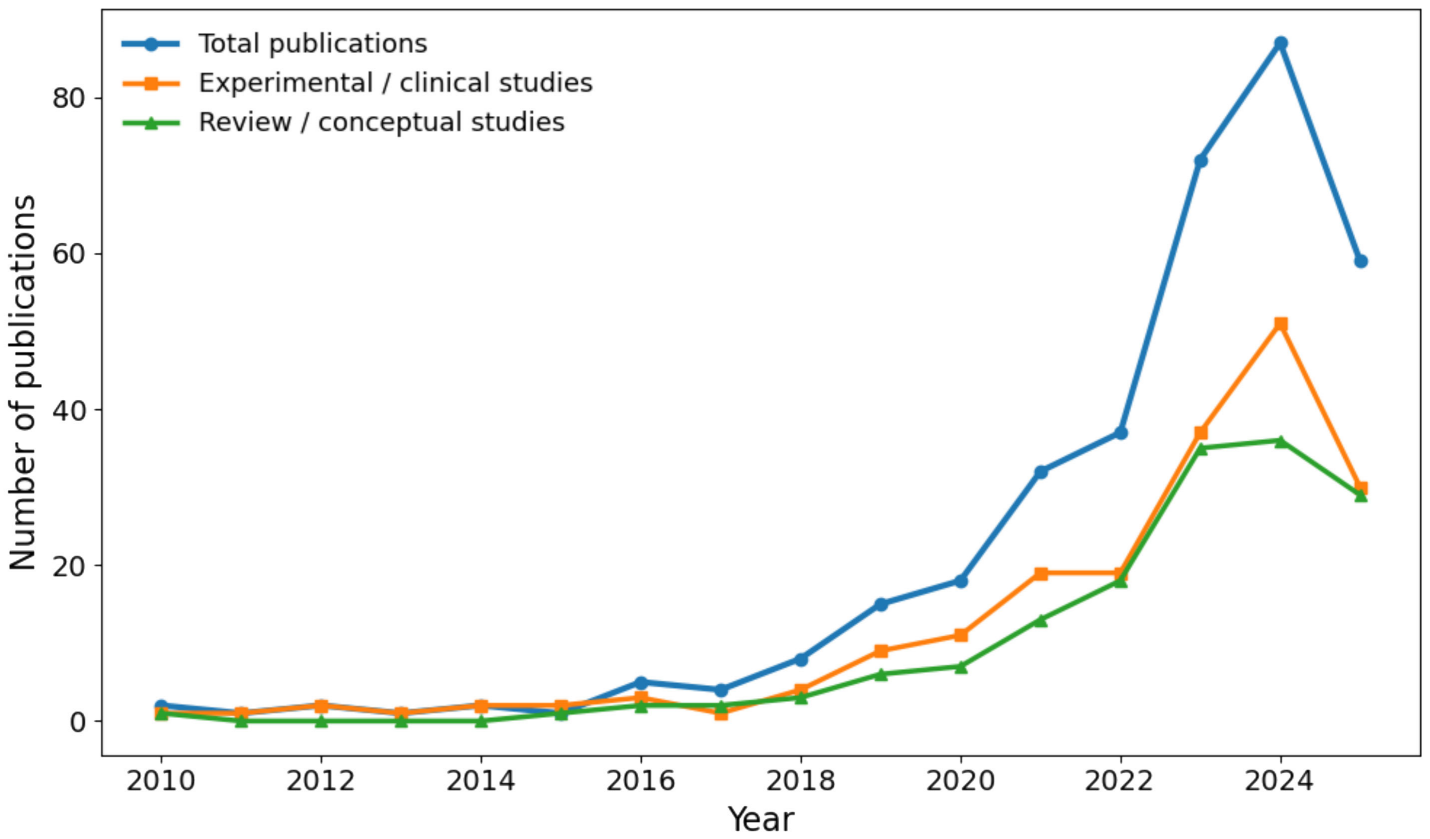
Temporal trends in DTP-related publications. Temporal trends in publications addressing drug-tolerant persister (DTP) cells in cancer from 2010 to 2025. The total number of publications is shown alongside the distribution of experimental/clinical studies and review or conceptual articles. The figure illustrates the rapid expansion of the field in recent years, with a marked increase in mechanistic and translational studies focused on early drug tolerance and adaptive resistance.

**Figure 3:**
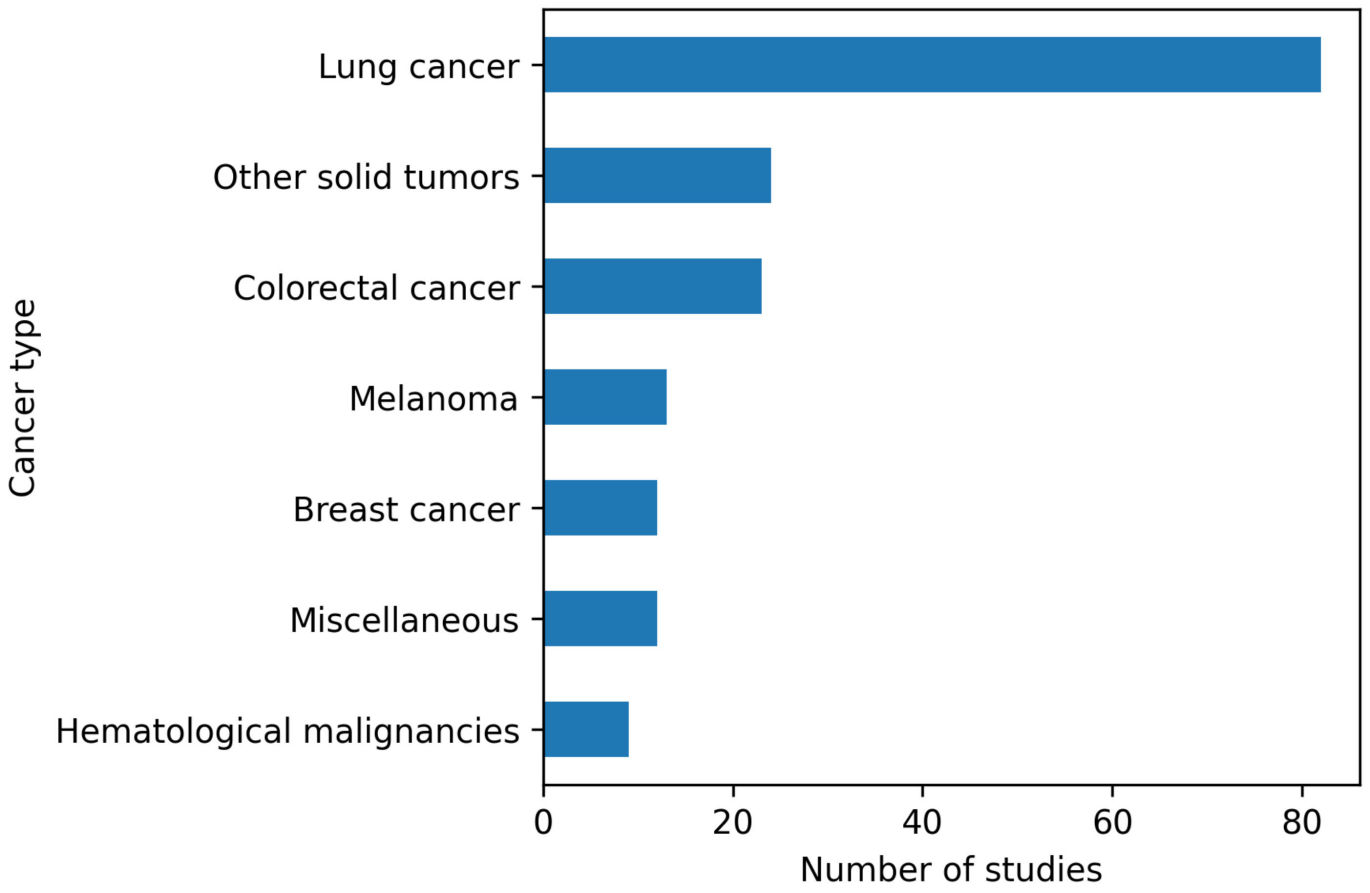
Distribution of cancer types investigated in experimental DTP studies. Distribution of cancer types represented in experimental studies investigating drug-tolerant persister (DTP) cells. Lung cancer models predominate, followed by colorectal cancer and a heterogeneous group of other solid tumors. Breast cancer, melanoma, hematological malignancies, and miscellaneous cancer types are less frequently represented, reflecting both experimental tractability and historical emphasis rather than the true prevalence of DTP biology across tumor types.

Quantitative analyses, including frequency distributions across experimental models, therapeutic categories, detection strategies, and molecular mechanisms, were performed using Python 3.11 in a Jupyter Notebook environment. Data processing and visualization relied on Pandas, NumPy, and Matplotlib. The PRISMA flow diagram (**Figure 1**), publication trends over time (**Figure 2**), and the distribution of cancer types across experimental studies (**Figure 3**) were generated directly from the curated dataset to ensure accuracy and reproducibility. The complete curated dataset underlying all quantitative analyses and figures is provided as **Supplementary Material**.

## 3 Results

### 3.1 Overview of the DTP cells in the literature landscape

Of the 770 records identified, 427 did not meet the eligibility criteria because they were exclusively computational, epidemiological, image-based, or otherwise irrelevant to the biological phenomenon of DTP cells. The records in the master database totaled 343 and met the inclusion criteria (**Figure 1**). After classification, 175 experimental or clinical-experimental studies and 168 review or conceptual articles were obtained.

The included literature spanned 2010–2025, demonstrating the rapid expansion of this field (**Figure 2**). DTP-related publications were relatively infrequent before 2016, with fewer than five articles per year. At the beginning of 2018, the number of studies increased steadily, rising from eight in 2018 to 15 in 2019 and 18 in 2020. A pronounced acceleration occurred between 2021 and 2024, rising from 32 articles in 2021 to 37 in 2022, followed by a substantial expansion to 72 in 2023, and 87 in 2024. Although 2025 was incomplete at the time of the search, 59 relevant records were published. These temporal patterns reflect the consolidation of DTP research as a central framework for understanding early therapeutic tolerance and its connection to acquired resistance (15, 16, 18).

Across experimental studies, lung cancer models predominated, followed by colorectal cancer and a heterogeneous group of other solid tumors, while breast cancer, melanoma, and hematological malignancies were less frequently represented (**Figure 3**). This distribution likely reflects both experimental tractability and historical emphasis on targeted therapy paradigms rather than the true prevalence of DTP biology across tumor types.

Experimental studies followed a similar tendency, increasing from 9 in 2019 to 13 in 2021, and expanding markedly in 2023 (37 articles) and 2024 (51 papers). Review articles exhibited comparable growth, surpassing 20 publications annually after 2023. Overall, these trends underscore the increasing recognition of DTP cells as a clinically relevant, non-genetic precursor to therapeutic resistance, contributing to minimal residual disease and enabling the emergence of stable resistant populations, as demonstrated in EGFR-mutant models and other systems (16, 28, 29).

### 3.2 Experimental models and functional definition of drug-tolerant persister cells

Across 175 experimental and experimental–clinical studies, several convergent methodological principles emerged despite substantial variations in cancer types, therapeutic agents, exposure conditions, and detection platforms. Together, these shared features support a functional definition of DTP cells applicable across diverse tumor models and experimental systems.

*In vitro* studies predominantly use two complementary approaches. The first involved acute high-dose drug exposure, typically at concentrations at or above the inhibitory concentration (IC) _50_ for 48–120 h. Under these conditions, most cells underwent apoptosis; however, a small subpopulation survived and exhibited canonical DTP characteristics, including reduced proliferation, apoptosis escape, and extensive transcriptional and metabolic reprogramming. The second approach relied on chronic continuous exposure to clinically relevant drug concentrations for 7–21 days. This strategy leads to population contraction, followed by the emergence of a slow-cycling, drug-tolerant fraction. Drug-withdrawal experiments have consistently demonstrated that these surviving cells regain their proliferative capacity upon removal of the therapeutic agent, confirming the reversible nature of this state and distinguishing DTP cells from clones with stable genetic resistance (14, 15, 30, 31). **Table 1** summarizes the representative experimental strategies across studies.

**Table 1:** Drug-tolerant persister (DTP) cells and experimental strategies for their induction. .

Model system	DTP-generation strategy	Exposure time	Criteria used to confirm DTP state	Representative studies
*In vitro* (2D cell lines)	Acute high-dose drug exposure (≥ IC_50_)	48–120 h	Residual viability; apoptosis escape	(15, 16, 32)
*In vitro* (2D cell lines)	Chronic continuous drug exposure	7–21 days	Reversibility after drug washout; slow-cycling phenotype	(16, 28, 29)
3D spheroids	Chronic exposure ± hypoxia	5–10 days	Reduced proliferation; metabolic rewiring; spatial survival pattern	(30, 33)
Organoids (patient-derived)	TKI/chemotherapy exposure; scRNA-seq detection of DTP clusters	5–14 days	DTP transcriptional cluster; partial EMT; survival under drug	(34, 35)
*In vivo* (PDX or residual disease)	Drug treatment until partial regression	Variable	Persistence after treatment; enrichment of tolerant clones	(14, 30, 36)

*In vivo* evidence has been obtained from xenograft and patient-derived xenograft (PDX) models, in which a small population of viable, drug-tolerant cells persisted despite treatment-induced tumor regression. The transcriptomic and chromatin accessibility profiles of these residual cell populations *in vivo* showed partial overlap with the *in vitro* DTP signatures, including enrichment of stress-response pathways and epigenetic remodeling programs. Clinical observations, including paired pre- and post-treatment biopsies and tumor fragments treated ex vivo with drugs, further corroborate the presence of DTP-like states in patient tumors (14, 30, 36).

Three-dimensional culture systems, including patient-derived spheroids and organoids, provide additional physiological evidence of DTP formation. Drug-treated spheroids typically develop spatially distributed DTP subpopulations that concentrate in hypoxic regions and exhibit a metabolic dependence on oxidative metabolism (e.g., OXPHOS) (17, 37). Studies using organoid models have revealed transcriptionally distinct clusters of DTP-like cells, marked by partial epithelial-mesenchymal transition (EMT), chromatin remodeling, and reduced biosynthetic activity (31, 36, 38). These populations have been identified using high-throughput approaches, including single-cell RNA sequencing (scRNA-seq), bulk RNA sequencing (bulk RNA-seq), and chromatin accessibility assays such as ATAC-seq (15, 39). Partial EMT states have been characterized by the co-expression of epithelial markers (e.g., EPCAM) and mesenchymal markers (e.g., VIM, ZEB1) (40), while chromatin remodeling has been inferred from dynamic changes in histone modifications and altered chromatin accessibility profiles (15, 39). Reduced biosynthetic activity has been associated with decreased expression of genes involved in ribosomal biogenesis, protein synthesis, and cell cycle progression (17, 41).

Emerging evidence suggests that residual tumor cell populations identified *in vivo* following treatment exhibit molecular characteristics that partially overlap with DTP states defined *in vitro*. Transcriptomic analyses of minimal residual disease and drug-treated tumors have revealed an enrichment of specific adaptive pathways, including the activation of stress response programs (e.g., ATF4/ISR signaling), epigenetic regulation mediated by histone-modifying enzymes (e.g., KDM5A-dependent chromatin remodeling), and survival signaling pathways such as the rewiring of PI3K/AKT and MAPK (15–17). Concurrently, these populations frequently exhibit states of partial epithelial-mesenchymal transition (EMT) and metabolic reprogramming toward oxidative phosphorylation (17, 40). It is worth noting that chromatin remodeling emerges as a central and dynamic feature of DTP biology, with studies reporting both globally repressive chromatin configurations associated with reduced transcriptional activity and, conversely, localized chromatin accessibility at loci related to stress response and survival (15, 39). These seemingly opposing states are not mutually exclusive but rather reflect a highly plastic and context-dependent epigenetic landscape that allows for selective gene expression under therapeutic stress. However, the *in vivo* context introduces additional regulatory layers, including microenvironmental influences such as hypoxia, immune interactions, and stromal signaling, which can further modulate these adaptive programs (8, 9). In summary, these observations support the concept that DTP cells represent a plastic and clinically relevant cellular state within a continuum linking transient drug tolerance to stable resistance.

Across *in vitro*, 3D culture, *in vivo*, and clinical systems, four criteria consistently define DTP cells as a reversible drug-tolerant state: survival of a minority subpopulation during high or prolonged drug exposure, recovery of proliferative capacity following drug withdrawal, reduced proliferation and apoptosis escape during treatment, and enhanced clonogenic potential enabling the eventual emergence of resistant outgrowth. Accordingly, drug-tolerant persister cells are best understood as residual cancer cells that survive therapeutic exposure through reversible, non-genetic adaptive mechanisms, enabling persistence under treatment and re-entry into the cell cycle once therapeutic pressure is removed. To facilitate comparison across tumor types, we summarized representative DTP-associated molecular markers and transcriptional signatures across major cancer contexts (**Table 2**).

**Table 2:** Representative molecular markers and transcriptional signatures associated with DTP cells across cancer types.

Cancer type	Experimental context	DTP-associated markers/signatures	Key features	Representative studies
NSCLC	EGFR TKI/chemotherapy	KDM5A↑, SOX2↑, AXL↑	Chromatin remodeling, stemness, survival signaling	(15, 16, 32)
Melanoma	BRAF inhibitors	AXL↑, NGFR↑, MITF↓	Phenotype switching, slow-cycling state	(14, 17)
Breast cancer	Chemotherapy/targeted therapy	ALDH1A1↑, OXPHOS↑	Metabolic reprogramming, stem-like features	(17, 41)
Colorectal cancer	Chemotherapy	p21↑, Ki67↓	Quiescence, reduced proliferation	(29, 32)
Pancreatic cancer	Chemotherapy/targeted therapy	OXPHOS↑, stress-response genes↑	Metabolic adaptation, survival under stress	(9, 35)

### 3.3 Molecular mechanisms that promote the emergence and survival of DTP cells

Despite methodological heterogeneity, experimental studies agree on an integrated signaling landscape that favors the emergence and survival of DTP cells. In all cancer types and model systems, DTP populations consistently rely on a coordinated network of reversible biological programs that enable them to survive under therapeutic pressure and facilitate their subsequent re-entry into the cell cycle upon treatment withdrawal.

Apoptotic escape is one of the most frequently observed characteristics of drug-tolerant persister cells. DTP cells exhibit reduced caspase activation, stabilization of mitochondrial outer membrane integrity, altered expression and balance of BCL2-family regulators, and suppression of pro-apoptotic signaling programs. Several studies have shown that DTP cells display increased dependence on anti-apoptotic proteins such as BCL-2, BCL-XL, or MCL-1, accompanied by reduced mitochondrial apoptotic priming. In addition, attenuation of p53-dependent transcriptional responses and suppression of death receptor signaling have been reported in tolerant populations, further limiting apoptotic commitment under drug pressure. Importantly, these features diminish following drug withdrawal, demonstrating that apoptosis evasion represents a reversible tolerance mechanism rather than a form of stable genetic resistance (36, 42, 43).

Reversible quiescence or slow cycling constitutes the second major adaptive program associated with drug-tolerant persister cells. Upon therapeutic exposure, DTP cells frequently enter a G_0_-like state characterized by markedly reduced proliferative activity, low or absent Ki-67 expression, and coordinated downregulation of core cell cycle regulators, including cyclin D1, CDK2, and E2F-dependent transcriptional programs. This cell cycle arrest is accompanied by profound transcriptional and metabolic rewiring, favoring stress tolerance over proliferation. DTP cells activate adaptive stress-response pathways such as the integrated stress response, exemplified by activation of the PERK–eIF2α axis and induction of ATF4-dependent transcriptional programs that promote amino acid homeostasis, redox balance, and survival under nutrient and drug-induced stress. In parallel, DTP cells enhance oxidative stress defenses through upregulation of antioxidant pathways, including NRF2-regulated genes involved in glutathione metabolism and reactive oxygen species detoxification. Autophagy-related programs are also frequently engaged, facilitating the recycling of intracellular components and sustaining metabolic flexibility during prolonged drug exposure. Concomitantly, DTP cells exhibit reduced DNA replication stress as a consequence of cell cycle exit, together with attenuated apoptotic priming mediated by shifts in BCL-2 family protein balance, thereby further enhancing tolerance to both cytotoxic agents and targeted therapies. Importantly, this quiescent or slow-cycling state remains reversible: upon drug withdrawal or relief of selective pressure, DTP cells can rapidly re-enter the cell cycle and restore proliferative capacity, clearly distinguishing them from senescent cells or terminally arrested populations that display stable growth arrest and irreversible phenotypic commitment (16, 29, 44).

The third central mechanism involves chromatin remodeling and epigenetic plasticity. ATAC-seq and ChIP-seq analyses revealed extensive reorganization of enhancer landscapes, redistribution of histone modifications, including H3K4me3 and H3K27ac, and altered transcription factor accessibility. Pharmacological inhibition of HDACs, KDMs, or BET proteins reverses these epigenetic states and restores drug sensitivity, demonstrating that DTP survival depends on dynamic and reversible chromatin configurations (15, 29, 30, 45, 46).

Phenotypic plasticity, particularly transitions involving hybrid epithelial–mesenchymal (E-M) states, also substantially contributes to DTP survival. These intermediate states promote cytoskeletal flexibility, alter adhesion dynamics, and activate survival signaling pathways. Inhibition of EMT-associated transcriptional programs markedly reduced DTP abundance across several model systems (30, 45, 46).

Metabolic rewiring is another prominent adaptive feature. DTP cells exhibited increased reliance on oxidative phosphorylation, elevated dependence on glutathione- and NADPH-mediated redox control, and preservation of mitochondrial respiratory fitness. Functionally, these metabolic vulnerabilities became evident when mitochondrial respiration was inhibited, such as by the blockade of complex I, which selectively impaired DTP survival. Similarly, disruption of glutathione-dependent antioxidant pathways or inhibition of mitophagy limits the ability of DTP cells to maintain redox homeostasis and mitochondrial quality control. Interference with lipid metabolic pathways, including the inhibition of fatty acid β-oxidation, similarly depletes DTP populations, underscoring metabolic plasticity as a fundamental vulnerability (43, 47, 48).

Within the broader experimental literature, two additional studies contributed to the characterization of early drug-tolerant persister states in lung adenocarcinoma models. Transcriptomic analyses following short-term exposure to cisplatin or EGFR-targeted therapies revealed that early DTP cells rapidly acquire non-proliferative, stress-adaptive transcriptional programs, including alterations in chromatin-associated regulators, metabolic pathways, and survival-related genes. Notably, these transcriptional changes emerged prior to stable genetic resistance and were associated with reversible drug tolerance, supporting the concept of DTP cells as an early, non-genetic adaptive state within the resistance continuum (49, 50).

Finally, the activation of alternative survival pathways provides an additional layer of protection. Signaling nodes such as AURKB, JNK, PI3Kγ, and FAK–YAP form compensatory circuits that maintain viability despite therapeutic inhibition of primary oncogenic pathways. Pharmacological targeting of these nodes reduced DTP abundance and delayed the emergence of stable resistant clones. For example, Aurora kinase A supports early survival and promotes the transition from drug tolerance to acquired resistance in EGFR-mutant models (51), whereas comprehensive mapping of osimertinib-tolerant persister cells identified actionable vulnerabilities across bypass pathways, including AURKB, PI3Kγ, and FAK–YAP signaling (29). Additional studies of stress-activated pathways, such as JNK-mediated cytoprotective signaling, have further demonstrated that the inhibition of these compensatory circuits can diminish DTP viability and suppress resistant outgrowth (52). Taken together, these findings describe DTP cells as a reversible and adaptive cellular state sustained by coordinated transcriptional, epigenetic, phenotypic, metabolic, and signaling pathways. These interdependent mechanisms collectively enable survival under therapeutic pressure and, if not eliminated, form the basis for subsequent resistant growth. **Table 3** summarizes the principal mechanistic categories identified in the studies.

**Table 3:** Molecular mechanisms supporting drug-tolerant persister (DTP) cell survival.

Mechanistic category	Core biological features	Representative evidence	Representative studies
Apoptosis escape	Reduced caspase activation; preserved mitochondrial integrity; BCL2/MCL1 upregulation	DTP cells survive strong apoptotic signaling and regain drug sensitivity after washout	(30, 36, 53)
Quiescence/slow cycling	Reversible G_0_-like arrest; low Ki-67 expression; slowed proliferation	Re-entry into the cell cycle following drug withdrawal	(16, 32, 54)
Chromatin remodeling	Altered H3K4me3 and H3K27ac marks; enhancer rewiring; transcription-factor accessibility changes	Epigenetic inhibitors reverse DTP-associated chromatin states	(15, 55, 56)
Phenotypic plasticity	Hybrid EMT states; YAP/FAK signaling; cytoskeletal remodeling	EMT or YAP/FAK inhibition reduces DTP abundance	(30, 35, 57)
Metabolic rewiring	Increased OxPhos; enhanced antioxidant defenses; mitophagy; lipid metabolism and FAO reliance	Targeting redox or mitochondrial metabolism selectively eliminates DTP cells	(54, 58, 59)
Signaling dependencies	AURKB, JNK, PI3Kγ, FAK–YAP compensatory survival circuits	Pathway inhibition reduces DTP viability and delays resistant outgrowth	(35, 45, 51)
Stemness programs	SOX2, ALDH1A1, CD44 enrichment; increased clonogenic capacity	Stem-like features diminish after drug removal	(28, 31, 55)

### 3.4 Therapeutic strategies targeting drug-tolerant persister cells

The growing recognition of DTP cells as key intermediates in the resistance continuum has prompted the development of therapeutic strategies to eliminate these resilient populations before the emergence of stable genetic resistance. Several pharmacological approaches have shown promising activity across diverse models by specifically targeting the adaptive programs that support DTP survival.

#### 3.4.1 Epigenetic therapies disrupting DTP maintenance

Epigenetic therapies, including HDAC, KDM, and BET inhibitors, disrupt the chromatin states required for DTP maintenance and synergize with targeted therapies to reduce DTP frequency (15, 55, 60). For example, the inhibition of histone deacetylases or demethylases interferes with the chromatin modifications that maintain DTP cells in a drug-tolerant state, such as the retention of closed or repressed chromatin at pro-apoptotic loci, thereby restoring susceptibility to drug-induced apoptosis (15, 55). BET inhibitors, which target bromodomain and extra-terminal domain proteins involved in enhancer regulation, also disrupt transcriptional programs that maintain DTP persistence under therapeutic pressure (60).

#### 3.4.2 Targeting metabolic and redox vulnerabilities

Metabolic and redox-targeting approaches exploit the increased dependence of DTP cells on oxidative phosphorylation and antioxidant defense systems. Inhibitors of mitochondrial complex I preferentially eliminate DTP cells by disrupting respiratory activity and reducing adenosine triphosphate (ATP) production. Interference with glutathione synthesis or other antioxidant defense pathways can increase intracellular ROS beyond tolerable thresholds, leading to selective DTP depletion (43, 48). Similarly, agents targeting lipid metabolism or fatty acid β-oxidation impair mitochondrial flexibility and reduce the survival of metabolically rewired DTP populations (54, 61).

#### 3.4.3 Inhibition of bypass survival signaling pathways

Inhibition of bypass survival pathways blocks compensatory signaling circuits that maintain DTP viability during drug exposure. Targeting pathways such as AURKB, PI3K–AKT, JNK, and FAK–YAP has been shown to prevent survival under therapeutic stress (52, 57, 62, 63). Pharmacological AURKB inhibition disrupts mitotic stress tolerance, whereas PI3K–AKT blockade prevents activation of alternative survival signals following primary oncogenic pathway inhibition. Inhibition of JNK signaling increases apoptosis and limits the emergence of TKI-tolerant cells, while interference with FAK–YAP signaling reduces adhesion- and cytoskeleton-mediated survival cues exploited by DTP (57).

#### 3.4.4 Strategies limiting phenotypic plasticity

Strategies designed to limit phenotypic plasticity have also demonstrated specific effects on DTP. Inhibitors of EMT-associated transcription factors or cytoskeletal remodelers can suppress the hybrid E-M states characteristic of DTP cells, sensitizing them to TKIs or chemotherapy, and reducing persistence (64–66). Targeting YAP/TAZ activity similarly disrupts plasticity-driven survival mechanisms (57).

#### 3.4.5 Stemness-directed interventions

Stemness-directed approaches, including inhibitors of SOX2-, ALDH-, or CD44-associated pathways, reduce clonogenic regrowth and limit the capacity of DTP cells to repopulate tumors after drug withdrawal (30, 31, 60). By impairing the transient stem-like programs induced during therapeutic tolerance, these interventions prevent the outgrowth of resistant populations.

#### 3.4.6 Combination strategies targeting DTP cells

The most promising therapeutic strategies involve combination regimens that integrate standard-of-care drugs with inhibitors targeting mechanisms associated with DTP. It has been demonstrated that the co-administration of chromatin-modifying agents with tyrosine kinase inhibitors (TKIs), or the combination of mitochondrial inhibitors with chemotherapy, reduces the number of surviving DTP cells and delays the emergence of stable resistant clones (16, 36, 60).

These findings support a therapeutic paradigm in which early intervention targeting DTP-associated adaptive programs could improve the durability of cancer therapies. To provide a translational context, we have summarized representative combination strategies targeting DTP-associated mechanisms in various cancer types, including their level of evidence (**Table 4**).

**Table 4:** Combination strategies targeting drug-tolerant persister (DTP)-associated mechanisms across cancer types and levels of evidence.

Target/mechanism	Combination strategy	Cancer type	Evidence level	Representative studies
Chromatin remodeling (KDM5A)	Epigenetic inhibitors targeting chromatin remodeling (e.g., KDM5, HDAC) + targeted therapy	NSCLC/multiple cancer types	Preclinical	(15, 39)
OXPHOS dependence	OXPHOS inhibition + targeted therapy/chemotherapy	Solid tumors/hematologic malignancies	Translational/preclinical	(17, 67)
AXL/EMT axis	AXL inhibitor + EGFR TKI	NSCLC	Early clinical/translational	(68, 69)
PI3K/AKT signaling	PI3K inhibitors + EGFR TKI or chemotherapy	NSCLC	Clinical/preclinical	(16)

## 4 Discussion

**Figure 4:**
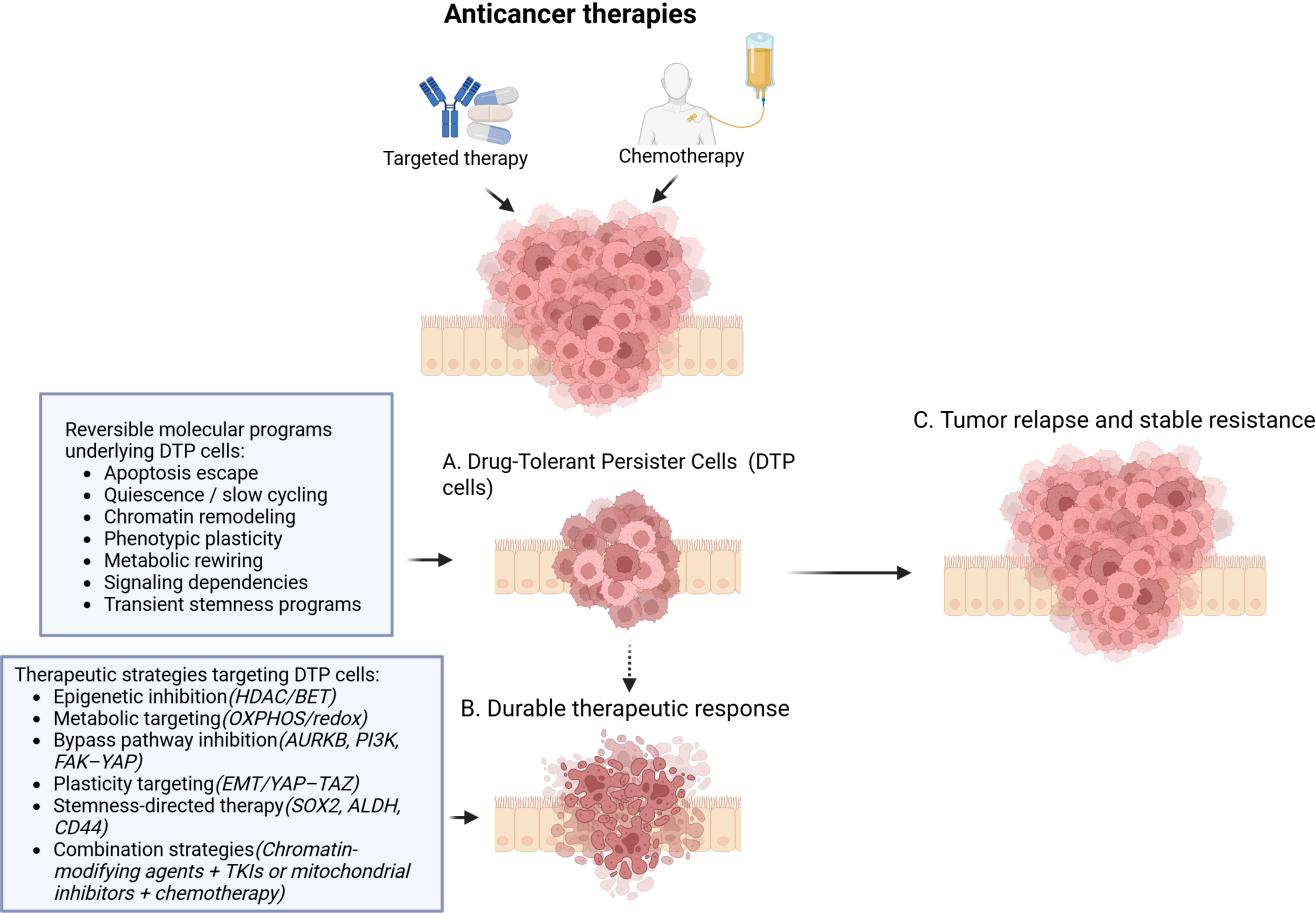
Reversible molecular programs and therapeutic strategies shaping DTP fate. Conceptual model illustrating the emergence and fate of drug-tolerant persister (DTP) cells following anticancer therapy. **(A)** Drug-tolerant persister state, characterized by a residual population of viable tumor cells that survive initial therapy through reversible, non-genetic adaptive programs. **(B)** Durable therapeutic response, achieved when DTP cells are effectively eliminated by DTP-targeting strategies, resulting in sustained suppression of tumor outgrowth. **(C)** Tumor relapse and stable resistance, arising when DTP cells persist under therapeutic pressure and subsequently give rise to genetically stable resistant clones that drive disease recurrence. Created in BioRender. Aguilar-Cazares, (2025) https://BioRender.com/5orkjmn.

The synthesis of 343 eligible records supports the view that drug-tolerant persister (DTP) cells represent a conserved and biologically significant adaptive state that arises across cancer types and therapeutic contexts. A central insight emerging from the integrated literature is that early survival following treatment is predominantly mediated by reversible, non-genetic adaptations rather than by the immediate acquisition of resistance-conferring mutations. This concept, first demonstrated in EGFR-mutant lung cancer models (15, 16), has since been extended to multiple solid and hematological malignancies, underscoring the generality of the DTP phenotype. These convergent adaptive programs and their impact on therapeutic outcomes are summarized in the conceptual framework presented in **Figure 4**.

In various experimental systems, including two-dimensional cultures, three-dimensional spheroids, organoids, xenografts, residual disease models, and patient-derived samples, DTP cells consistently share three defining properties: the ability to survive high or prolonged exposure to drugs, entry into a reversible slow-cycling or inactive state, and recovery of proliferative capacity and drug sensitivity after treatment withdrawal. Although there is substantial heterogeneity in experimental design, drug dosing, and detection criteria, such variability primarily affects quantitative estimates rather than the qualitative reproducibility of the DTP state.

From the perspective of molecular mechanisms, DTP survival does not depend on a single dominant pathway but reflects the coordinated participation of multiple adaptive programs. These include suppression of apoptotic priming, reversible reprogramming of transcription and chromatin remodeling, partial epithelial-mesenchymal transition, and broader phenotypic plasticity. Metabolic adaptations are a particularly prominent feature, characterized by increased reliance on oxidative phosphorylation, enhanced redox homeostasis, and preservation of mitochondrial integrity through quality control mechanisms such as mitophagy. In parallel, DTP cells exploit compensatory signaling circuits, including pathways such as AURKB, JNK, PI3Kγ, and FAK-YAP, which remain active when major oncogenic drivers such as EGFR, BRAF, or MEK are inhibited (15, 16, 29). The transient enrichment of stem cell-like characteristics further contributes to the regenerative potential of DTP cells following drug withdrawal. Taken together, these mechanisms position DTP cells as a reversible adaptive state that may precede or facilitate the emergence of stable genetic resistance. Although DTP cells are primarily defined by reversible, non-genetic adaptations (15), several studies have described increased genomic instability and mutational processes in persistent cell populations subjected to prolonged drug exposure (16). It is important to note that these alterations are generally interpreted as a consequence of the tolerance state, rather than as the initiating mechanism of DTP formation. Over time, these processes may facilitate the transition from transient drug tolerance to genetically stable resistance, supporting the existence of a continuum between these phenotypes (11).

DTP states can arise under different therapeutic exposure paradigms, including acute high-dose treatment and chronic, continuous drug exposure. Although these conditions differ in duration and selective pressure, several studies have reported partially overlapping molecular programs between these states, including activation of stress-response pathways, partial epithelial-to-mesenchymal transition (EMT), metabolic reprogramming, and chromatin remodeling (15, 17). However, important differences also exist, as chronic exposure may allow for more sustained adaptation and increased genomic instability over time (16, 64). These observations suggest that DTP cells generated under distinct treatment conditions may represent related but not identical phenotypic states within a broader adaptive continuum of therapy resistance.

Importantly, understanding the molecular pathways and mechanisms involved in the emergence of DTP cells has direct therapeutic implications. Multiple studies demonstrate that targeting chromatin regulators, metabolic dependencies, redox homeostasis, or downstream survival signaling can selectively impair DTP viability and delay the emergence of acquired resistance. These findings support combined strategies that integrate standard therapies with drugs targeting mechanisms associated with DTP cells, as summarized in **Table 4**, and underscore the importance of early intervention approaches to reduce the reservoir of cells capable of triggering a relapse.

Several limitations of this exploratory review should be acknowledged. Despite a comprehensive search strategy across multiple databases, variability in indexing practices may have resulted in the omission of recently published or emerging studies. Furthermore, the diversity of experimental models and methodological approaches limits the feasibility of quantitative synthesis and complicates direct comparisons between studies. Although evidence from *in vivo* models and clinical samples is growing, most mechanistic insights continue to derive from *in vitro* or ex vivo systems, which may limit translational generalizability. Finally, given the rapid expansion of the field, relevant findings beyond the temporal scope of this review are likely to continue to emerge.

Overall, the accumulated evidence supports a model in which DTP cells occupy an early and reversible position within a broader continuum of resistance. By persisting under therapeutic pressure, DTP cells create a reservoir of viable cells with the potential for stress-associated mutagenesis and clonal evolution, ultimately enabling stable resistance and disease relapse. Recognizing DTP cells as a reproducible and treatable adaptive state has important implications for therapeutic innovation, monitoring of minimal residual disease, and the rational design of strategies aimed at preventing cancer recurrence.

## 5 Conclusion

DTP cells represent a reversible and adaptive survival state that enables cancer cells to withstand therapeutic pressure and contributes to the emergence of stable genetic resistance. By integrating 343 studies from diverse model systems, cancer types, and therapeutic contexts, this exploratory review defines the fundamental phenotypic and molecular characteristics of DTP cells, describes the experimental strategies used for their identification, and synthesizes the major adaptive programs that underpin their survival.

The convergence of chromatin remodeling, metabolic rewiring, apoptosis escape, slow cell cycle, phenotypic plasticity, and stemness-associated traits highlights the complex yet addressable nature of this cellular state.

Taken together, these findings position DTP cells as a central component of the early adaptive response to treatment and a critical intermediary within the continuum of cancer resistance. Targeting DTP-associated mechanisms represents a promising avenue for eliminating minimal residual disease, delaying or preventing relapse, and improving the long-term efficacy of cancer therapies.

## Supplementary material

Supplementary Table 1 Curated dataset of the 343 studies included in this scoping review. The table contains information on cancer type, experimental model, treatment category, DTP detection strategy, proposed molecular mechanisms, validation approaches, and study classification.

## References

[1] Papac RJ . Origins of cancer therapy. Yale J Biol Med. (2001) 74:391–8. PMC2588755 11922186

[2] Goodman LS . Nitrogen mustard therapy: Use of methyl-bis(beta-chloroethyl)amine hydrochloride and tris(beta-chloroethyl)amine hydrochloride for Hodgkin’s disease, lymphosarcoma, leukemia and certain allied and miscellaneous disorders. JAMA. (1984) 251:2255. doi: 10.1001/jama.1984.03340410063036. PMID: 20997191

[3] DeVita VT Chu E . A history of cancer chemotherapy. Cancer Res. (2008) 68:8643–53. doi: 10.1158/0008-5472.CAN-07-6611. PMID: 18974103

[4] Soria J-C Ohe Y Vansteenkiste J Reungwetwattana T Chewaskulyong B Lee KH . Osimertinib in untreated EGFR -mutated advanced non–small-cell lung cancer. N Engl J Med. (2018) 378:113–25. doi: 10.1056/NEJMoa1713137. PMID: 29151359

[5] Chapman PB Hauschild A Robert C Haanen JB Ascierto P Larkin J . Improved survival with vemurafenib in melanoma with BRAF V600E mutation. N Engl J Med. (2011) 364:2507–16. doi: 10.1056/NEJMoa1103782. PMID: 21639808 PMC3549296

[6] Baselga J Cortés J Kim S-B Im S-A Hegg R Im Y-H . Pertuzumab plus trastuzumab plus docetaxel for metastatic breast cancer. N Engl J Med. (2012) 366:109–19. doi: 10.1056/NEJMoa1113216. PMID: 22149875 PMC5705202

[7] Phi LTH Sari IN Yang Y-G Lee S-H Jun N Kim KS . Cancer stem cells (CSCs) in drug resistance and their therapeutic implications in cancer treatment. Stem Cells Int. (2018) 2018:1–16. doi: 10.1155/2018/5416923. PMID: 29681949 PMC5850899

[8] Niu X Liu W Zhang Y Liu J Zhang J Li B . Cancer plasticity in therapy resistance: Mechanisms and novel strategies. Drug Resist Update. (2024) 76:101114. doi: 10.1016/j.drup.2024.101114. PMID: 38924995

[9] Ramisetty S Subbalakshmi AR Pareek S Mirzapoiazova T Do D Prabhakar D . Leveraging cancer phenotypic plasticity for novel treatment strategies. J Clin Med. (2024) 13:3337. doi: 10.3390/jcm13113337. PMID: 38893049 PMC11172618

[10] Marine J-C Dawson S-J Dawson MA . Non-genetic mechanisms of therapeutic resistance in cancer. Nat Rev Cancer. (2020) 20:743–56. doi: 10.1038/s41568-020-00302-4. PMID: 33033407

[11] França GS Baron M King BR Bossowski JP Bjornberg A Pour M . Cellular adaptation to cancer therapy along a resistance continuum. Nature. (2024) 631:876–83. doi: 10.1038/s41586-024-07690-9. PMID: 38987605 PMC11925205

[12] Russo M . Genetic and non‐genetic drug resistance: Darwin or Lamarck? Mol Oncol. (2024) 18:241–4. doi: 10.1002/1878-0261.13601. PMID: 38308461 PMC10850810

[13] Davies A Zoubeidi A Beltran H Selth LA . The transcriptional and epigenetic landscape of cancer cell lineage plasticity. Cancer Discov. (2023) 13:1771–88. doi: 10.1158/2159-8290.CD-23-0225. PMID: 37470668 PMC10527883

[14] Roesch A Fukunaga-Kalabis M Schmidt EC Zabierowski SE Brafford PA Vultur A . A temporarily distinct subpopulation of slow-cycling melanoma cells is required for continuous tumor growth. Cell. (2010) 141:583–94. doi: 10.1016/j.cell.2010.04.020. PMID: 20478252 PMC2882693

[15] Sharma SV Lee DY Li B Quinlan MP Takahashi F Maheswaran S . A chromatin-mediated reversible drug-tolerant state in cancer cell subpopulations. Cell. (2010) 141:69–80. doi: 10.1016/j.cell.2010.02.027. PMID: 20371346 PMC2851638

[16] Hata AN Niederst MJ Archibald HL Gomez-Caraballo M Siddiqui FM Mulvey HE . Tumor cells can follow distinct evolutionary paths to become resistant to epidermal growth factor receptor inhibition. Nat Med. (2016) 22:262–9. doi: 10.1038/nm.4040. PMID: 26828195 PMC4900892

[17] Hangauer MJ Viswanathan VS Ryan MJ Bole D Eaton J Schreiber SL . Drug-tolerant persister cancer cells are vulnerable to GPX4 inhibition. Cancer Res. (2017) 77(7679):247–50. doi: 10.1158/1538-7445.AM2017-1006. PMID: 29088702 PMC5933935

[18] Russo M Chen M Mariella E Peng H Rehman SK Sancho E . Cancer drug-tolerant persister cells: From biological questions to clinical opportunities. Nat Rev Cancer. (2024) 24:694–717. doi: 10.1038/s41568-024-00737-z. PMID: 39223250 PMC12622869

[19] Hata AN Larijani M . Targeting APOBECs in cancer: It’s about timing. Cancer Cell. (2024) 42:497–501. doi: 10.1016/j.ccell.2024.03.010. PMID: 38593778 PMC12160365

[20] Bigger JW . Treatment of staphylococcal infections with penicillin by intermittent sterilisation. Lancet. (1944) 244:497–500. doi: 10.1016/S0140-6736(00)74210-3. PMID: 37496192

[21] Balaban NQ Merrin J Chait R Kowalik L Leibler S . Bacterial persistence as a phenotypic switch. Science. (2004) 305:1622–5. doi: 10.1126/science.1099390. PMID: 15308767

[22] Lewis K . Persister cells, dormancy and infectious disease. Nat Rev Microbiol. (2007) 5:48–56. doi: 10.1038/nrmicro1557. PMID: 17143318

[23] Brauner A Fridman O Gefen O Balaban NQ . Distinguishing between resistance, tolerance and persistence to antibiotic treatment. Nat Rev Microbiol. (2016) 14:320–30. doi: 10.1038/nrmicro.2016.34. PMID: 27080241

[24] Tricco AC Lillie E Zarin W O'Brien KK Colquhoun H Levac D . PRISMA Extension for Scoping Reviews (PRISMA-ScR): Checklist and Explanation. Ann Intern Med. (2018) 169:467–73. doi: 10.7326/M18-0850, PMID: 30178033

[25] Peters MDJ Marnie C Tricco AC Pollock D Munn Z Alexander L . Updated methodological guidance for the conduct of scoping reviews. JBI Evid Implement. (2021) 19:3–10. doi: 10.1097/XEB.0000000000000277, PMID: 33570328

[26] Higgins JPT Thomas J Chandler J Cumpston M Li T Page MJ . Cochrane handbook for systematic reviews of interventions. London: Cochrane (2022). Available online at: https://training.cochrane.org/handbook (Accessed October 8, 2025).

[27] Arksey H O’Malley L . Scoping studies: towards a methodological framework. Int J Soc Res Methodol. (2005) 8:19–32. doi: 10.1080/1364557032000119616

[28] Kashima Y Shibahara D Suzuki A Muto K Kobayashi IS Plotnick D . Single-cell analyses reveal diverse mechanisms of resistance to EGFR tyrosine kinase inhibitors in lung cancer. Cancer Res. (2021) 81:4835–48. doi: 10.1158/0008-5472.CAN-20-2811. PMID: 34247147 PMC8448980

[29] Criscione SW Martin MJ Oien DB Gorthi A Miragaia RJ Zhang J . The landscape of therapeutic vulnerabilities in EGFR inhibitor osimertinib drug tolerant persister cells. NPJ Precis Oncol. (2022) 6(1):95. doi: 10.1038/s41698-022-00337-w. PMID: 36575215 PMC9794691

[30] Glasheen MQ Caksa S Young AG Wilski NA Ott CA Chervoneva I . Targeting upregulated cIAP2 in SOX10-deficient drug tolerant melanoma. Mol Cancer Ther. (2023) 22:1087–99. doi: 10.1158/1535-7163.MCT-23-0025. PMID: 37343247 PMC10527992

[31] Nojima Y Yao R Suzuki T . Single-cell RNA sequencing and machine learning provide candidate drugs against drug-tolerant persister cells in colorectal cancer. Biochim Biophys Acta Mol Basis Dis. (2025) 1871:167693. doi: 10.1016/j.bbadis.2025.167693. PMID: 39870146

[32] Oren Y Tsabar M Cuoco MS Amir-Zilberstein L Cabanos HF Hütter J-C . Cycling cancer persister cells arise from lineages with distinct programs. Nature. (2021) 596:576–82. doi: 10.1038/s41586-021-03796-6. PMID: 34381210 PMC9209846

[33] Han GYQ Alexander M Gattozzi J Day M Kirsch E Tafreshi N . Ecological and evolutionary dynamics to design and improve ovarian cancer treatment. Clin Transl Med. (2024) 14:e70012. doi: 10.1002/ctm2.70012. PMID: 39210542 PMC11362027

[34] Álvarez-Varela A Novellasdemunt L Barriga FM Hernando-Momblona X Cañellas-Socias A Cano-Crespo S . Mex3a marks drug-tolerant persister colorectal cancer cells that mediate relapse after chemotherapy. Nat Cancer. (2022) 3:1052–70. doi: 10.1038/s43018-022-00402-0. PMID: 35773527

[35] Pfeifer M Brammeld JS Price S Pilling J Bhavsar D Farcas A . Genome-wide CRISPR screens identify the YAP/TEAD axis as a driver of persister cells in EGFR mutant lung cancer. Commun Biol. (2024) 7(1):497. doi: 10.1038/s42003-024-06190-w. PMID: 38658677 PMC11043391

[36] Watanabe H Nakagomi H Hirotsu Y Amemiya K Mochizuki H Inoue M . TP53-positive clones are responsible for drug-tolerant persister and recurrence of HER2-positive breast cancer. Breast Cancer Res Treat. (2022) 196:255–66. doi: 10.1007/s10549-022-06731-z. PMID: 36087189

[37] Tixier F Hatt M Valla C Fleury V Lamour C Ezzouhri S . Visual versus quantitative assessment of intratumor 18F-FDG PET uptake heterogeneity: prognostic value in non-small cell lung cancer. J Nucl Med. (2014) 55:1235–41. doi: 10.2967/jnumed.113.133389. PMID: 24904113

[38] Dart A . Cycling persister cells. Nat Rev Cancer. (2021) 21:683–. doi: 10.1038/s41568-021-00409-2. PMID: 34522021

[39] Knoechel B Roderick JE Williamson KE Zhu J Lohr JG Cotton MJ . An epigenetic mechanism of resistance to targeted therapy in T cell acute lymphoblastic leukemia. Nat Genet. (2014) 46:364. doi: 10.1038/ng.2913. PMID: 24584072 PMC4086945

[40] Pastushenko I Brisebarre A Sifrim A Fioramonti M Revenco T Boumahdi S . Identification of the tumour transition states occurring during EMT. Nature. (2018) 556:463–8. doi: 10.1038/s41586-018-0040-3. PMID: 29670281

[41] Vallette FM Olivier C Lezot F Oliver L Cochonneau D Lalier L . Dormant, quiescent, tolerant and persister cells: Four synonyms for the same target in cancer. Biochem Pharmacol. (2019) 162:169–76. doi: 10.1016/j.bcp.2018.11.004. PMID: 30414937

[42] Giron P Eggermont C Noeparast A Vandenplas H Teugels E Forsyth R . Targeting USP13-mediated drug tolerance increases the efficacy of EGFR inhibition of mutant EGFR in non-small cell lung cancer. Int J Cancer. (2021) 148:2579–93. doi: 10.1002/ijc.33404. PMID: 33210294 PMC8048518

[43] Deepak K Roy PK Das CK Mukherjee B Mandal M . Mitophagy at the crossroads of cancer development: Exploring the role of mitophagy in tumor progression and therapy resistance. Biochim Biophys Acta BBA - Mol Cell Res. (2024) 1871:119752. doi: 10.1016/j.bbamcr.2024.119752. PMID: 38776987

[44] Thiruvalluvan M Billet S Liu Z Lownik J Waissengrin B Kim H . CD105 blockade restores osimertinib sensitivity in drug-resistant EGFR-mutant non-small cell lung cancer. Drug Resist Update. (2025) 81:101237. doi: 10.1016/j.drup.2025.101237. PMID: 40090182

[45] Böpple K Oren Y Henry WS Dong M Weller S Thiel J . ATF3 characterizes aggressive drug-tolerant persister cells in HGSOC. Cell Death Dis. (2024) 15(4):290. doi: 10.1038/s41419-024-06674-x. PMID: 38658567 PMC11043376

[46] Hariri A Mirian M Khosravi A Zarepour A Iravani S Zarrabi A . Intersecting pathways: The role of hybrid E/M cells and circulating tumor cells in cancer metastasis and drug resistance. Drug Resist Update. (2024) 76:101119. doi: 10.1016/j.drup.2024.101119. PMID: 39111134

[47] Wang S Chang Y Liu T Huang K Fang W Li AF . Mitochondrial dysfunction decreases cisplatin sensitivity in gastric cancer cells through upregulation of integrated stress response and mitokine GDF15. FEBS J. (2023) 291:1131–50. doi: 10.1111/febs.16992. PMID: 37935441

[48] Lypova N Dougherty SM Clem BF Feng J Yin X Zhang X . PFKFB3-dependent redox homeostasis and DNA repair support cell survival under EGFR-TKIs in non-small cell lung carcinoma. Cancer Metab. (2024) 12:37. doi: 10.1186/s40170-024-00366-y. PMID: 39696407 PMC11658331

[49] Chavez-Dominguez R Aguilar-Cazares D Perez-Medina M Avila-Rios S Soto-Nava M Mendez-Tenorio A . Transcriptional signature of early cisplatin drug-tolerant persister cells in lung adenocarcinoma. Front Oncol. (2023) 13:1208403. doi: 10.3389/fonc.2023.1208403. PMID: 37916165 PMC10616253

[50] Perez-Medina M Lopez-Gonzalez JS Benito-Lopez JJ Ávila-Ríos S Soto-Nava M Matias-Florentino M . Transcriptomic analysis reveals early alterations associated with intrinsic resistance to targeted therapy in lung adenocarcinoma cell lines. Cancers. (2024) 16:2490. doi: 10.3390/cancers16132490. PMID: 39001552 PMC11240825

[51] Shah KN Bhatt R Rotow J Rohrberg J Olivas V Wang VE . Aurora kinase A drives the evolution of resistance to third-generation EGFR inhibitors in lung cancer. Nat Med. (2019) 25:111. doi: 10.1038/s41591-018-0264-7. PMID: 30478424 PMC6324945

[52] Tanimura K Yamada T Horinaka M Katayama Y Fukui S Morimoto K . Inhibition of c-Jun N-terminal kinase signaling increased apoptosis and prevented the emergence of ALK-TKI-tolerant cells in ALK-rearranged non-small cell lung cancer. Cancer Lett. (2021) 522:119–28. doi: 10.1016/j.canlet.2021.09.018. PMID: 34534615

[53] Tanaka K Yu HA Yang S Han S Selcuklu SD Kim K . Targeting Aurora B kinase prevents and overcomes resistance to EGFR inhibitors in lung cancer by enhancing BIM- and PUMA-mediated apoptosis. Cancer Cell. (2021) 39:1245–1261.e6. doi: 10.1016/j.ccell.2021.07.006. PMID: 34388376 PMC8440494

[54] Rehman SK Haynes J Collignon E Brown KR Wang Y Nixon AML . Colorectal cancer cells enter a diapause-like DTP state to survive chemotherapy. Cell. (2021) 184:226–242.e21. doi: 10.1016/j.cell.2020.11.018. PMID: 33417860 PMC8437243

[55] Vinogradova M Gehling VS Gustafson A Arora S Tindell CA Wilson C . An inhibitor of KDM5 demethylases reduces survival of drug-tolerant cancer cells. Nat Chem Biol. (2016) 12:531. doi: 10.1038/nchembio.2085. PMID: 27214401

[56] Tian Y Bhattacharya R Yoo S Jiang F Park E Lara Granados G . Epigenomic analysis identifies DTP subpopulation using HOPX to develop targeted therapy resistance in lung adenocarcinoma. iScience. (2025) 28(5):112387. doi: 10.1016/j.isci.2025.112387. PMID: 40352726 PMC12063144

[57] Shi R Farnsworth DA Febres-Aldana CA Chow JLM Sheena R Atwal T . Drug tolerance and persistence to EGFR inhibitor treatment are mediated by an ILK-SFK-YAP signaling axis in lung adenocarcinoma. Oncogene. (2025) 44(32):2831–49. doi: 10.1038/s41388-025-03461-6. PMID: 40450112 PMC12318776

[58] Li Y Chen H Xie X Yang B Wang X Zhang J . PINK1-mediated mitophagy promotes oxidative phosphorylation and redox homeostasis to induce. Cancer Res. (2023) 83:398–413. doi: 10.1158/0008-5472.CAN-22-2370. PMID: 36480196

[59] Tau S Chamberlin MD Yang H Marotti JD Muskus PC Roberts AM . Oxidative phosphorylation is a metabolic vulnerability of endocrine therapy-tolerant persister cells in ER^+^ breast cancer. Cancer Res. (2025) 85:1145–61. doi: 10.1158/0008-5472.CAN-24-1204. PMID: 39777474 PMC11908958

[60] Lee J Mashima T Kawata N Yamamoto N Morino S Inaba S . Pharmacologic Targeting of Histone H3K27 Acetylation/BRD4-dependent Induction of ALDH1A3 for Early-phase Drug Tolerance of Gastric Cancer. Cancer Res Commun. (2024) 4:1307–20. doi: 10.1158/2767-9764.CRC-23-0639, PMID: 38669046 PMC11104289

[61] Lin H Wang L Chen H Shen Y Wang C Xue Y . Mitochondrial fatty acid oxidation as the target for blocking therapy-resistance and inhibiting tumor recurrence: The proof-of-principle model demonstrated for ovarian cancer cells. J Adv Res. (2025) 79:571–85. doi: 10.1016/j.jare.2025.03.026. PMID: 40107354 PMC12766189

[62] Tetsu O Hangauer MJ Phuchareon J Eisele DW McCormick F . Drug resistance to EGFR inhibitors in lung cancer. Chemotherapy. (2016) 61:223–5. doi: 10.1159/000443368, PMID: 26910730 PMC4844777

[63] Wang C Liu J Wu Y Cai C Chai Z Jia P . AURKB as a therapeutic target and key driver of liver cancer growth and metastasis. APMIS. (2025) 133:e70021. doi: 10.1111/apm.70021. PMID: 40177797

[64] Nilsson MB Sun H Robichaux J Pfeifer M McDermott U Travers J . A YAP/FOXM1 axis mediates EMT-associated EGFR inhibitor resistance and increased expression of spindle assembly checkpoint components. Sci Transl Med. (2020) 12. doi: 10.1126/scitranslmed.aaz4589. PMID: 32878980 PMC8269000

[65] Nilsson MB Yang Y Heeke S Patel SA Poteete A Udagawa H . CD70 is a therapeutic target upregulated in EMT-associated EGFR tyrosine kinase inhibitor resistance. Cancer Cell. (2023) 41:340–355.e6. doi: 10.1016/j.ccell.2023.01.007. PMID: 36787696 PMC10259078

[66] Tokumo K Masuda T Nakashima T Namba M Yamaguchi K Sakamoto S . Association between plasminogen activator inhibitor-1 and osimertinib tolerance in EGFR-mutated lung cancer via epithelial-mesenchymal transition. Cancers. (2023) 15. doi: 10.3390/cancers15041092. PMID: 36831438 PMC9954529

[67] Khalaf A De Beauchamp L Kalkman E Rattigan K Himonas E Jones J . Nutrient-sensitizing drug repurposing screen identifies lomerizine as a mitochondrial metabolism inhibitor of chronic myeloid leukemia. Sci Transl Med. (2024) 16:eadi5336. doi: 10.1126/scitranslmed.adi5336. PMID: 38865484

[68] Park K Chang G-C Curigliano G Lim W-T Soo RA Molina-Vila MA . Phase I results of S49076 plus gefitinib in patients with EGFR TKI-resistant non-small cell lung cancer harbouring MET/AXL dysregulation. Lung Cancer. (2021) 155:127–35. doi: 10.1016/j.lungcan.2021.03.012. PMID: 33798902

[69] He K Berz D Gadgeel SM Iams WT Bruno DS Blakely CM . MRTX-500 phase 2 trial: Sitravatinib with nivolumab in patients with nonsquamous NSCLC progressing on or after checkpoint inhibitor therapy or chemotherapy. J Thorac Oncol. (2023) 18:907–21. doi: 10.1016/j.jtho.2023.02.016. PMID: 36842467 PMC10330304

